# How Synthetic Biology and Metabolic Engineering Can Boost the Generation of Artificial Blood Using Microbial Production Hosts

**DOI:** 10.3389/fbioe.2018.00186

**Published:** 2018-11-30

**Authors:** August T. Frost, Irene H. Jacobsen, Andreas Worberg, José L. Martínez

**Affiliations:** ^1^Section of Synthetic Biology, Department of Biotechnology and Biomedicine Technical University of Denmark (DTU), Lyngby, Denmark; ^2^Novo Nordisk Foundation Center for Biosustainability Technical University of Denmark (DTU), Lyngby, Denmark

**Keywords:** synthetic biology, metabolic engineering, yeast, protein production, blood substitutes, HBOC, recombinant hemoglobin

## Abstract

Hemoglobin is an essential protein to the human body as it transports oxygen to organs and tissues through the bloodstream (Looker et al., 1992). In recent years, there has been an increasing concern regarding the global supply of this vital protein, as blood availability cannot currently meet the high demands in many developing countries. There are, in addition, several risks associated with conventional blood transfusions such as the presence of blood-borne viruses like HIV and Hepatitis. These risks along with some limitations are presented in Figure 1 (Kim and Greenburg, 2013; Martínez et al., 2015). As an alternative, producing hemoglobin recombinantly will eliminate the obstacles, since hemoglobin-based oxygen carriers are pathogen-free, have a longer shelf-life, are universally compatible and the supply can be adjusted to meet the demands (Chakane, 2017). A stable, safe, and most importantly affordable production, will lead to high availability of blood to the world population, and hence reduce global inequality, which is a focus point of the World Health Organization for the millennium (WHO, 2018). Synthetic biology and metabolic engineering have created a unique opportunity to construct promising candidates for hemoglobin production (Liu et al., 2014; Martínez et al., 2016). This review sets out to describe the recent advances in recombinant hemoglobin production, the societal and the economic impact along with the challenges that researchers will face in the coming years, such as low productivity, degradation, and difficulties in scale-up. The challenges are diverse and complex but with the powerful tools provided by synthetic biology and metabolic engineering, they are no longer insurmountable. An efficient production of cell-free recombinant hemoglobin poses tremendous challenges while having even greater potential, therefore some possible future directions are suggested in this review.

**Figure 1:**
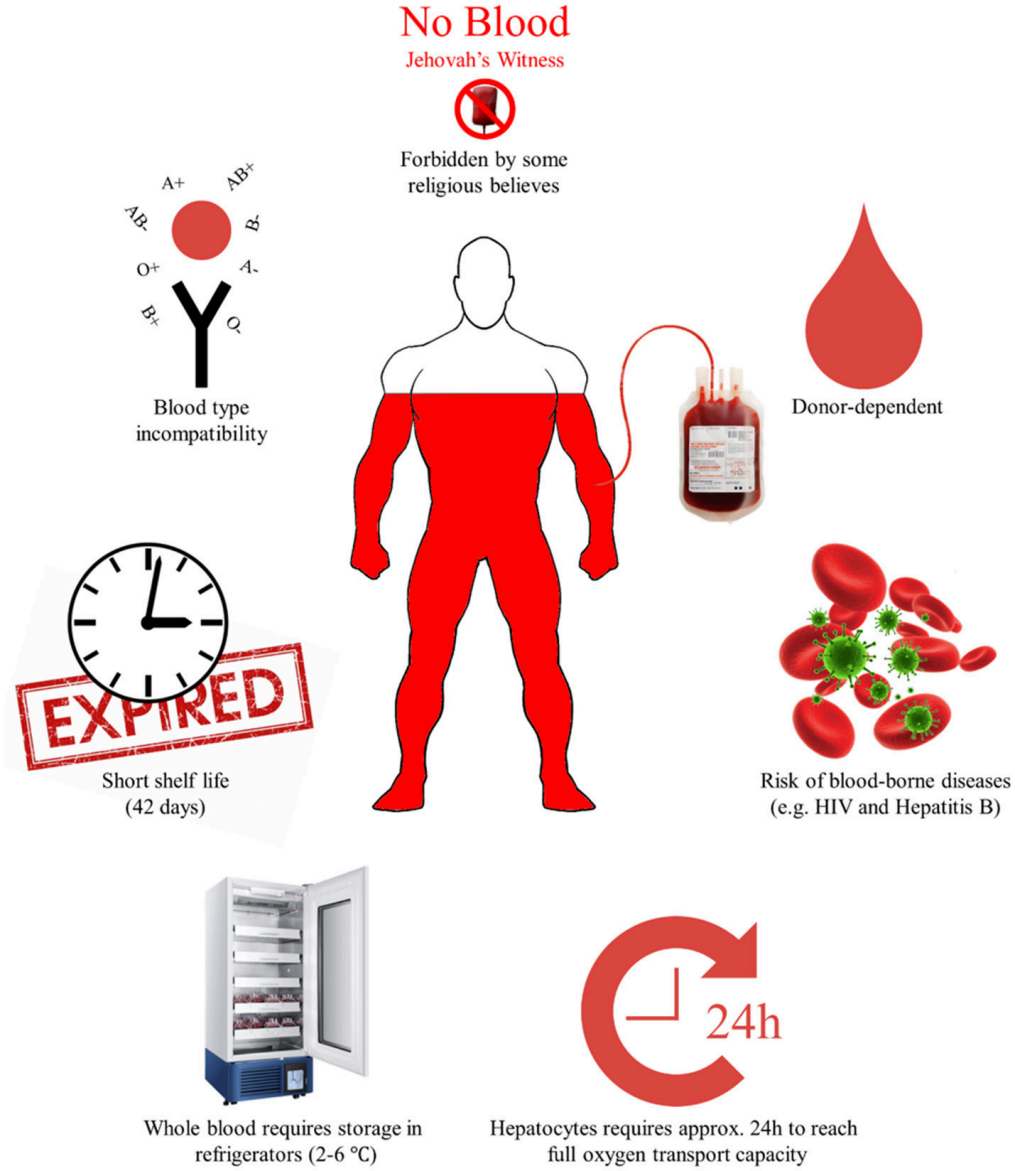
The limitations to conventional blood transfusions. The limitations are divided into three distinct groups; (a) limitations in storage and shelf life, (b) limitations due to donor dependency, blood-borne diseases, and incompatibility of blood types, and (c) limitations due to religious beliefs.

## Historical Perspective—Societal and Medical Needs

Since the first recorded blood transfusion in the ancient Inca civilization (fifteenth century), there have been countless attempts to create alternative blood substitutes to further improve the chance of surviving anemia (Sarkar, 2008). In the dawn of this development, elementary liquids such as sheep blood, urine, and beer were tested for the possibility to be a substitute for human blood. In the late nineteenth century, even cow milk was injected into patients with the belief that it would help regenerate white blood cells (Chen et al., 2009).

The earliest report of a hemoglobin-based blood substitute originates from the beginning of the 1930s, however it was not until 1957 that the first study on an artificial red blood cell composed by microencapsulated hemoglobin was performed (Li et al., 2005). After a stagnant period, the advancement was significantly stimulated by the onset of AIDS, hepatitis, and HIV infections in the 1980s, which imposed a radical risk to conventional blood transfusions (Kim and Greenburg, 2013; Martínez et al., 2015). Consequently, the following period was a golden age for the development of artificial substitutes that would be able to mimic the oxygen-carrying property of erythrocytes (Varnado et al., 2013; Martínez et al., 2015).

Through history, the pursuit of artificial blood substitutes has been heavily funded by the military to eliminate the supply, storage, and portability problems associated with human blood. However, the societal and medical need to find an alternative to conventional blood transfusion has escalated in recent years, especially in developing countries (Kim and Greenburg, 2013; Moradi et al., 2016). This is due to factors such as population growth, natural disasters, decreasing donor number, population aging, terror attacks, and the risk of blood-borne pathogens threatening the supply-demand balance of human blood (Looker et al., 1992; Varnado et al., 2013; Alayash, 2014; Moradi et al., 2016; Chakane, 2017).

The development of blood substitutes furthermore has the potential to get rid of logistical barriers for pre-hospital use in acute emergency situations and remote civilian locations, by enabling long-term storage, eliminating blood type matching, supplying adequate quantities, be “pathogen-free,” and providing immediate availability in catastrophic scenarios (Chakane, 2017).

## Recent Advances

Recent breakthroughs in the fields of synthetic biology and metabolic engineering have substantially boosted the development of the so-called oxygen carriers, which can be divided into two main categories: perfluorocarbon-based substitutes (PFC) and hemoglobin-based oxygen carriers (HBOCs) (Mozafari et al., 2015). While the research in the PFC as blood substitutes has been partially discontinued, due to their limited oxygen transfer capacity to tissues and toxicity problems, the capacity of HBOCs to mimic red blood cells in terms of oxygen transport while being less toxic, has put the focus on them. Hemoglobin supply is, therefore, the first key issue for the successful development of the latter. The primary sources of hemoglobin for the current development of HBOCs are expired human blood, mammalian hemoglobins formed as a by-product in the meat industry (e.g., bovine hemoglobin), and recombinant hemoglobin produced in transgenic organisms (Motwani et al., 1996; Sanders et al., 1996; Varnado et al., 2013; Alayash, 2014; Moradi et al., 2016). The two first options are not the optimal choices, as their availability is too limited to allow a substantial development in HBOCs research. It is paramount for the case of the recombinant hemoglobin production, that new production hosts are designed with an increased and more efficient production capacity. Here, synthetic biology and metabolic engineering are key role players, both in terms of designing better cell factories and achieving affordable production costs.

*Escherichia coli* was the first choice as production workhorse. On a first attempt, a single β-globin chain with a cleavable linker was expressed, and subsequently refolded *in vitro* with a native α-globin chain and exogenous heme (Nagai and Thøgersen, 1987). In a follow-up experimental setup, α-globin and β-globin chains were co-expressed *in vivo* with endogenous heme incorporated (Shen et al., 1993). Although recombinant hemoglobin was successfully produced in bacteria, later advances focused on eukaryal hosts, as a result of the discovery that the vital functions of the hemoglobin produced by bacterial hosts were altered, most likely due to the methionine termini at the end of the globin chains. The alterations were identified to be caused by reduced Bohr effect and 2,3-BPG effects of the produced recombinant hemoglobin compared to normal human hemoglobin (Hoffman et al., 1990).

Thus, the production shifted to the model yeast *Saccharomyces cerevisiae*. Since then, synthetic biology and metabolic engineering have been applied to modify the heme biosynthetic pathway by overexpressing the genes coding for enzymes responsible for the rate-limiting steps (e.g., *HEM3*). Furthermore, the optimal globin expression balance between α and β chains was studied. In combination, the recombinant hemoglobin production was improved by an impressive 87% (Liu et al., 2014) compared to the previous attempts.

Important key issues to take into consideration, in order to get further in the advancement of HBOCs, apart from the sustained hemoglobin supply, are the safety concerns and cost-effectiveness. Non-clinical and clinical studies of HBOCs have raised safety concerns regarding hemoglobin extravasation across the blood vessel wall, scavenging of endothelial nitric oxide, oversupply of oxygen, and oxidative side reactions (Alayash, 2014). As a result, the regulatory agencies in the United States and the European Union have not yet approved any HBOCs (Chen et al., 2009; Varnado et al., 2013; Mozafari et al., 2015; Meng et al., 2018).

## Economical Perspective and Societal Impact

A study performed in the United States have estimated that it is currently possible to produce recombinant human hemoglobin at a cost of approximately $11/g. However, if the operating expenses and equipment investments are included, the cost increases to a dazzling amount of ≥$200/g. In comparison, human hemoglobin can be derived from alternative sources for roughly $2/g to $4/g (excl. post-related costs) based on fixed reimbursement prices of whole blood packs (Varnado et al., 2013).

There is, consequently, a demanding need to significantly improve the cost-effectiveness by either reducing the production cost or increasing the expression yield of recombinant hemoglobin by more than 3-fold to be cost-effective against alternatives. Synthetic biology can contribute with further optimizations to provide affordable recombinant hemoglobin at a comparable price to alternatives in the future. WHO has created a set of goals to improve global health for the world population by 2020—these are called “*HEALTH 2020”* (WHO, 2018). Recombinant hemoglobin is extremely relevant to one of the goals, which is to reduce inequality in the access to the medicine of the twenty first century and save lives.

## The Challenges of Recombinant Hemoglobin Production

The evolution of synthetic biology has given researchers the possibility to synthesize new genetic elements instead of transferring them from a donor organism (Stephanopoulos, 2012). Nowadays entire genomes can be synthesized and inserted into a host organism (Kuo et al., 2018). Biotechnological research has also benefited from synthetic biology, as it synergizes with metabolic engineering. Synthetic biology provides genetic switches, vectors, characterized enzymes, and minimal hosts, all of which minimizes the cost and the time needed for metabolic engineering (Keasling, 2012).

**Figure 2:**
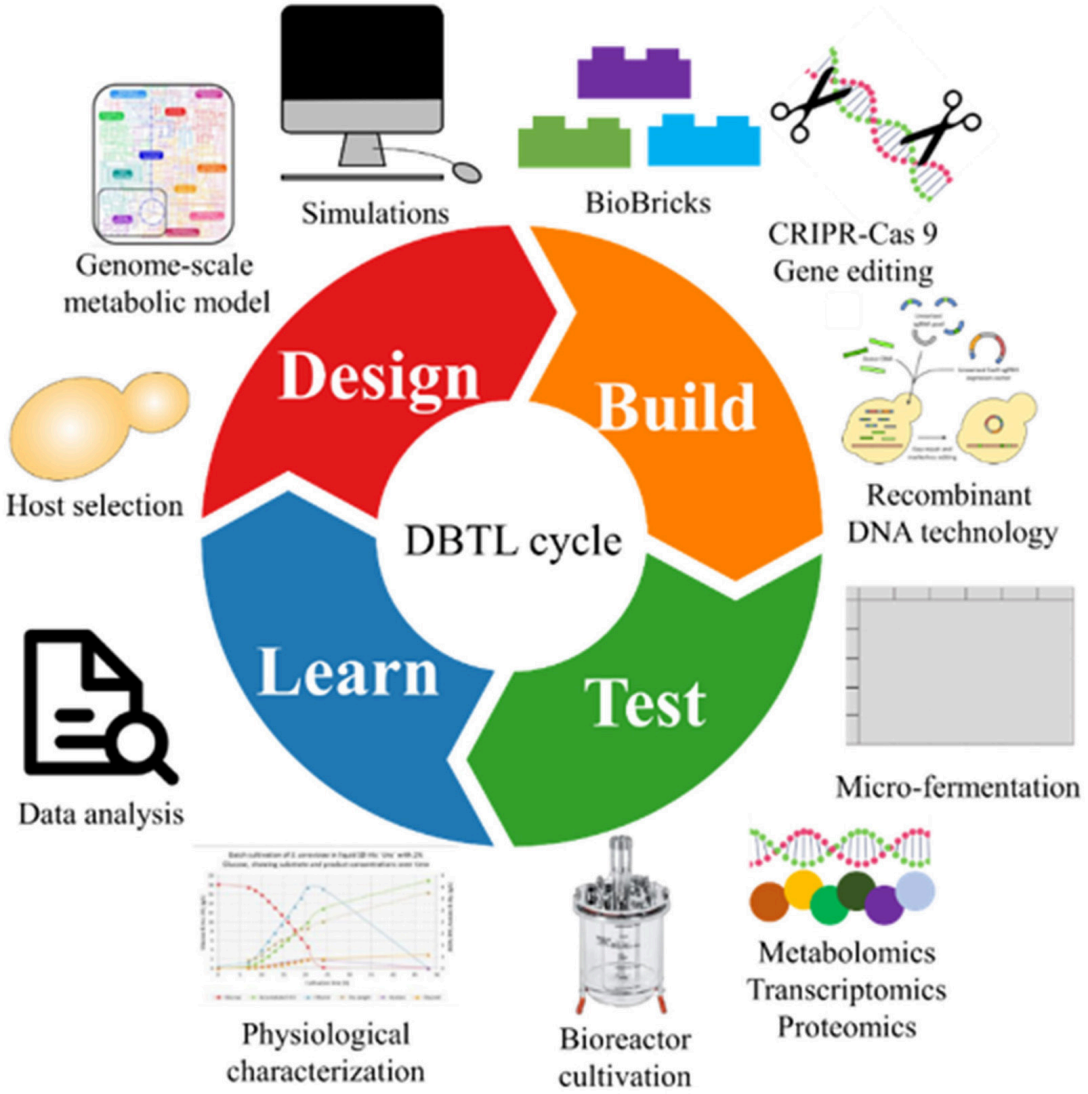
The Design-Build-Test-Learn cycle (DBTL-cycle). The “Design”-phase includes setting goals, choosing a host organism, using models and simulations, and planning the design of the organism. In the “Build”-phase the organism is constructed based on the planned design. The “Test”-phase covers the in-depth testing of the organism. The final phase is the “Learn”-phase where the results are analyzed and compared to the predetermined specifications.

To successfully engineer novel cell factories a number of frameworks can be applied, one of the most popular is the “*Design-Build-Test-Learn”* cycle—Figure 2. The cycle is an iterative process divided into four phases, which aims at satisfying a set of predetermined specifications. The first phase is the “*Design”*-phase where the organism is designed *in silico*. That design is then constructed in the “*Build”*-phase. The third phase is the “*Test”*-phase, where the organism or construct is tested in terms of productivity, -*omics*, and growth characteristics. The final part is the “*Learn”*-phase, where the results from the “*Test”-*phase are evaluated and compared to the predetermined specifications set in the “*Design”-*phase. Often multiple iterations of the cycle are needed to achieve the optimal organism and satisfy the specifications (Ando and Martin, 2018; Jensen and Keasling, 2018). Synthetic biology and metabolic engineering contribute mainly to the first two phases (Nielsen and Keasling, 2016).

### Higher Productivity

As previously described, the production of recombinant hemoglobin from a heterologous host is not yet economically feasible. Without accounting for operation cost and the expenses of establishing the production plant, the estimated costs should still be reduced by at least three-fold before being able to compete on the market. One of the challenges is low productivity. In 2016, Martìnez et al. developed a recombinant *S. cerevisiae*-based cell factory. After a few iterations of the DBTL cycle, the constructed stain was able to synthesize functional human hemoglobin with α-globin and β-globin genes expressed in the optimal ratios, being the maximum measured concentration of active human hemoglobin up to 7% of the total cell protein content (Martínez et al., 2016). In perspective, this means that this strain would be able to produce 200 kg of hemoglobin in a 100 m^3^ tank, assuming the highest theoretical yield. Considering that one liter of human blood contains 150 grams of hemoglobin, the equivalent to 1.300 liters of blood would be produced in one batch. These are, however, ideal conditions in which upscaling limitations and recovery of the product are not considered. To reach these theoretical numbers at production-scale, a higher productivity needs to be achieved in laboratory-scale. Enhancing productivity in a heterologous organism is a task in the “*Design”*-phase of the DBTL-cycle. Classically a lot of work would have to be conducted, and a lot of manpower required, in order to plan and test hypotheses which might lead to higher productivity. In recent years, both the number and the variety of accurate open-end genome-scale models have increased (Nielsen and Keasling, 2016). Applying these models to the rational design can save time in the optimization of the productivity. It is now possible to find a robust, open-end genome-scale model of *S. cerevisiae*, that can be customized to have the genetic modifications implemented in previous research. Different strategies can be executed, and a simulation will reveal the success of the strategy in terms of enhanced productivity. Bottlenecks and feedback inhibitions can be identified and hence eliminated (Nielsen and Keasling, 2016). Ultimately, only successful *in-silico* strategies will be carried out experimentally.

### Intracellular Degradation and Negative Feedback Circuits

In the pursuit of higher productivities, most of the times undesired trade-offs are found as a consequence. Normally, degradation of precursors due to inherent feedback regulatory reactions is the main issue, and recombinant hemoglobin production is, unfortunately, not an exception to this matter. Heme is essential to the yeast cell for signaling and co-factor purposes, but it is also cytotoxic in higher concentrations as it can originate reactive oxygen species (ROS). To prevent accumulation and thereby toxicity, heme biosynthesis is tightly controlled and coordinated with heme degradation in well-understood processes (Martínez et al., 2016; Hanna et al., 2018). When heme is bound in hemoglobin, ROS are no longer formed, and heme will not be degraded (Liu et al., 2014).

A rapidly forming hemoglobin would therefore remove un-bound heme from the mitochondrion and hence stop degradation. Thus, the key is to have an abundance of α and β chains ready for assembly. In the “*Test”-*phase, proteomics combined with the advances in mass spectrometry, can assist the quantification of the chains and thereby identify possible limitations in the following “*Learn”-*phase (Trent, 2012). Other ways to reduce degradation involve gene knock-outs. For instance, genes involved in (i) iron-depletion initiated degradation of heme to recover the iron (Philpott and Protchenko, 2008) (ii) vacuolar miss-sorting and vacuolar degradation of mature hemoglobin (Ammerer et al., 1986; Marcusson et al., 1994; Hecht et al., 2014). Gene edition is tedious and very limiting most of the times, however the introduction and refining of the CRISPR-Cas9 system can drastically shorten the duration of the “*Build”*-phases where multiple knock-outs or insertions are needed. The method allows for gene disruption in up to five different loci in *S. cerevisiae* with a high success rate. The method is, moreover, marker-free (Jakočiunas et al., 2015).

### Oxygen Limitation and Scale-Up

Hemoglobin synthesis in yeast is induced by high levels of extracellular oxygen (Martínez et al., 2016). In production-scale vessels adequate oxygen supply and transfer pose an issue due to the low solubility of oxygen in submerged cultures. To ensure a linear scale-up of oxygen availability other parameters need to be modified, e.g., agitation which can lead to shear stress (Reuss, 2008; Garcia-Ochoa and Gomez, 2009). Switching the point of view from process engineering to genetic and metabolic engineering could solve the problem. In 2015, Martìnez et al. deleted a heme activator protein (*HAP1*) in *S. cerevisiae* and found that heme production was increased due to the removal of a feedback inhibition. Another measured effect of the *HAP1* mutant is the reduction of respiration (Martínez et al., 2015). In the ideal scenario a large-scale production could be established based on a non-respiring strain where the presence of oxygen would initiate heme synthesis. The feasibility of the strategy can be predicted based on model-simulations rather than real-life cultivations in production scale.

### Ethanol Production

The Crabtree-positive nature of *S. cerevisiae* (meaning its ability to produce ethanol from glucose in aerobic conditions) is one of the challenges in recombinant hemoglobin production for several reasons: (i) lower yields as carbon is diverted into ethanol production (Dai et al., 2018) and (ii) yields of hemoglobin are lower on ethanol than on glucose (Liu et al., 2014). The code to engineering a Crabtree-negative *S. cerevisiae* strain has finally been cracked by a Dai et al. who used state-of-the-art synthetic biology and molecular biology tools. The group reverse engineered a strain which had evolved under Adapted Laboratory Evolution. The created strain is the first Crab-negative *S. cerevisiae* reported to be able to grow at high glucose concentrations (Dai et al., 2018).

This particular strain in combination with the strategies stated above could create the future *S. cerevisiae*-based hemoglobin producer. Some challenges could arise as the Crabtree negative *S. cerevisiae* needs mitochondrial respiration to reoxidize co-factors, whereas it is beneficial to knock-out *HAP1* to avoid respiration in recombinant hemoglobin production (Martínez et al., 2015; Dai et al., 2018). Simulations with an open-end genome-scale model will disclose whether further design is needed on co-factor reoxidizing or compartmentalization.

### Recovery and Purification

Recovery and purification of hemoglobin from *S. cerevisiae* differs from the challenges described above, as it requires a new iteration of the DBTL-cycle. All the recombinant hosts designed to date, store the produced hemoglobin intracellularly. To recover the intracellular hemoglobin numerous unit operations will be required (Bulmer et al., 1996; Stocker-Majd et al., 2008). Here we will not discuss on how to solve the task by process engineering, but rather the solutions that can be created by using synthetic biology and metabolic engineering.

The rational approach sounds simple: to secrete the hemoglobin and recover it from the fermentation broth. All though *S. cerevisiae* is a desired platform for heterologous protein production, the yeast only secretes a few of its native proteins. To successfully secrete hemoglobin the protein needs the correct signal peptide, and genes involved in protein translocation should be upregulated (Tang et al., 2015; Bao et al., 2017). The combined powers of synthetic biology and metabolic engineering can decrease the amount of time and experiments needed. Combinations of genes and signal peptides can be modeled using genome-scale models to find the optima. The material for the modifications can be synthetically generated or taken directly from a BioBrick library (Constante et al., 2011) and lastly, CRIPSR-Cas9 can serve as an efficient and marker-free method for gene insertions or deletions (Jakočiunas et al., 2015). However, it is a complex task to make multimeric proteins secreted. Synthetic biology can contribute through the design of novel protein structures that would facilitate secretion, and hence the downstream processing, while keeping the functional properties intact. As an example, there are currently several strategies ongoing in order to design fusion hemoglobin molecules as a single peptide, but keeping a domain-like structure that resembles the multimeric structure.

### Novel Production Hosts

Experimenting with novel organisms is no longer just a wish from the researchers: new technologies have made the wish come true. With BioBricks and minimal hosts, organisms can be constructed and customized like mechanical objects—such as airplanes (Constante et al., 2011; Keasling, 2012). BioBricks are standardized which means that they are modular and can be assembled in any way desired, and can be ordered from the Standard Registry of Biological Parts which is a growing collection (Constante et al., 2011). It is not farfetched to envision that a paradigm shift will occur in the near future of biotechnology. Researchers will be able to design the organism that they want instead of working around native shortcomings.

All of these technologies can widen the array of possible cell factories. For the production of recombinant hemoglobin this means that the production platform will no longer be restricted to *S. cerevisiae*. The performance of more organisms can be tested out both theoretically (using models) and experientially (using well-defined modular components) to find the optimal one. With the recent advances in high through-put micro-fermentation systems all-new stains can be designed, constructed and tested rapidly (Kensy et al., 2009).

## Concluding Remarks

The implementation and continuous development of synthetic biology and metabolic engineering tools have enabled researchers to move the boundaries of what cell factories can do. The tools cannot only be used for creating new “super-drugs” but also to make well-known treatments more available.

Producing hemoglobin recombinantly in yeast has proven feasible. The challenges which still remain are considered solvable with the combined forces of knowledge, synthetic biology and metabolic engineering. This review has described some of the possible strategies to further improve yeast-based cell factories.

Currently, commercial production of recombinant hemoglobin is too expensive, to match the price of blood-derived hemoglobin. The cost should be further decreased by three-fold, but once strains have been optimized and large production plants have been established, hemoglobin can be produced at large scale. Yeasts are easy to cultivate and require cheap substrates in order to produce hemoglobin. It is also important to note that recombinant hemoglobin is not associated with the same risks as blood- derived hemoglobin in terms of pathogens and unstable supply.

All in all, the production of recombinant hemoglobin can ensure access to safe and affordable blood substitutes for the entire world population. Ultimately, the production of recombinant hemoglobin via synthetic biology will decrease inequality and help reach the goals set by WHO.

## Author Contributions

AF, IJ, and JM wrote the manuscript. AW and JM edited and supervised the final version.

### Conflict of Interest Statement

The authors declare that the research was conducted in the absence of any commercial or financial relationships that could be construed as a potential conflict of interest.
